# Predictors of late arrhythmic events after generator replacement in Brugada syndrome treated with prophylactic ICD

**DOI:** 10.3389/fcvm.2022.964694

**Published:** 2022-07-22

**Authors:** Federico Migliore, Nicolò Martini, Leonardo Calo', Annamaria Martino, Giulia Winnicki, Riccardo Vio, Chiara Condello, Alessandro Rizzo, Alessandro Zorzi, Luigi Pannone, Vincenzo Miraglia, Juan Sieira, Gian-Battista Chierchia, Antonio Curcio, Giuseppe Allocca, Roberto Mantovan, Francesca Salghetti, Antonio Curnis, Emanuele Bertaglia, Manuel De Lazzari, Carlo de Asmundis, Domenico Corrado

**Affiliations:** ^1^Department of Cardiac, Thoracic, Vascular Sciences and Public Health, University of Padova, Padova, Italy; ^2^Department of Cardiology, Policlinico Casilino, Rome, Italy; ^3^Heart Rhythm Management Centre, Postgraduate Program in Cardiac Electrophysiology and Pacing, Universitair Ziekenhuis Brussel-Vrije Universiteit Brussel, European Reference Networks Guard-Heart, Brussels, Belgium; ^4^Division of Cardiology, Department of Medical and Surgical Sciences, Magna Graecia University, Catanzaro, Italy; ^5^Department of Cardiology, S.Maria dei Battuti Hospital, Conegliano, Italy; ^6^Spedali Civili Hospital, University of Brescia, Brescia, Italy

**Keywords:** Brugada syndrome, implantable cardioverter-defibrillator, risk stratification, sudden cardiac death, complications

## Abstract

**Introduction:**

Predictors of late life-threatening arrhythmic events in Brugada syndrome (BrS) patients who received a prophylactic ICD implantation remain to be evaluated. The aim of the present long-term multicenter study was to assess the incidence and clinical-electrocardiographic predictors of late life-threatening arrhythmic events in BrS patients with a prophylactic implantable cardioverter defibrillator (ICD) and undergoing generator replacement (GR).

**Methods:**

The study population included 105 patients (75% males; mean age 45 ± 14years) who received a prophylactic ICD and had no arrhythmic event up to first GR.

**Results:**

The median period from first ICD implantation to last follow-up was 155 (128–181) months and from first ICD Implantation to the GR was 84 (61–102) months. During a median follow-up of 57 (38–102) months after GR, 10 patients (9%) received successful appropriate ICD intervention (1.6%/year). ICD interventions included shock on ventricular fibrillation (*n* = 8 patients), shock on ventricular tachycardia (*n* = 1 patient), and antitachycardia pacing on ventricular tachycardia (*n* = 1 patient). At survival analysis, history of atrial fibrillation (log-rank test; *P* = 0.02), conduction disturbances (log-rank test; *P* < 0.01), S wave in lead I (log-rank test; *P* = 0.01) and first-degree atrioventricular block (log-rank test; *P* = 0.04) were significantly associated with the occurrence of late appropriate ICD intervention. At Cox-regression multivariate analysis, S-wave in lead I was the only independent predictor of late appropriate ICD intervention (HR: 9.17; 95%CI: 1.15–73.07; *P* = 0.03).

**Conclusions:**

The present study indicates that BrS patient receiving a prophylactic ICD may experience late appropriate intervention after GR in a clinically relevant proportion of cases. S-wave in lead I at the time of first clinical evaluation was the only independent predictor of persistent risk of life-threatening arrhythmic events. These findings support the need for GR at the end of service regardless of previous appropriate intervention, mostly in BrS patients with conduction abnormalities.

## Introduction

Risk stratification and management of patients with Brugada syndrome (BrS), principally asymptomatic, still remain challenging (1–6). Many prognostic markers have been proposed, such as male gender, spontaneous type 1 BrS ECG pattern, positive electrophysiological study (EPS), fever, and resting situation (7–13). The role of genetic on risk stratification has been questioned. However, specific genetic mutations may be predictive, such as the combination of a SCN5A mutation with malignant arrhythmic events, ECG conduction abnormalities and the extent of the electrophysiological abnormalities (14, 15). In addition, ECG and imaging markers (16) seem to be useful for risk stratification. The predictive value of these parameters is based on non-invasive assessment of depolarization and repolarization parameters (17), such as: prolongation of PR interval (18), increase of QRS duration (19), fragmented QRS (f-QRS) (20, 21), S-wave in lead I (22), prolongation of QT interval or early repolarization (ER) pattern (23, 24). Even today, the implantable cardioverter-defibrillator (ICD) is the mainstay of treatment of BrS patients, although it is associated with high complication rates, including inappropriate shocks (IS) and lead failure (1–3, 25–27). Thus, when considering ICD implantation in BrS patients the risk/benefit balance should be considered. The subset of BrS patients requiring generator replacement (GR) who received a prophylactic ICD and did not experience appropriate interventions during the life of the first implantation rises challenging problems of management. Data on the arrhythmic outcome and predictors of late life-threatening arrhythmic events in this unique group of BrS patients after GR are incompletely established. The aim of the present long-term multicenter study was to assess the incidence and clinical-electrocardiographic predictors of late life-threatening arrhythmias in BrS patients treated with prophylactic ICD and undergoing GR.

## Methods

The study population included BrS patients, with spontaneous or drug induced Type 1 ECG pattern who received a prophylactic ICD either transvenous ICD (TV-ICD) or subcutaneous ICD (S-ICD) and had no arrhythmic event up to first GR. The patients were enrolled at six centers (Cardiology Department of the University of Padova, Hospital of Conegliano, the University of Brescia, the University Hospital of Catanzaro, the Casilino Hospital of Rome and the Universitair Ziekenhuis Brussel), between January 1996 and September 2020. Herein, an ICD was defined as “prophylactic” when it was implanted in patients without prior sustained ventricular tachycardia (VT) or ventricular fibrillation (VF) who were considered at high risk of sudden cardiac death (SCD) on the basis of current recognized risk factors (1–10). In the case of a transvenous defibrillator implant, the type of venous access (subclavian, axillary, or cephalic vein), the type of lead fixation (passive or active), and the type of device (single or dual chamber) were at the discretion of the physician. Furthermore, the choice of implanting an S-ICD rather than a TV-ICD was also at discretion of the physician, according to current guidelines (28). Brugada syndrome was diagnosed as previously reported (1, 2). Provocative drug test using ajmaline (1 mg/kg in 5–10 min) or flecainide (2 mg/kg in 5 min) was administered intravenously to unmask the diagnostic ECG pattern of BrS in case of a non-diagnostic baseline electrocardiogram (1, 2). Family history of BrS, or sudden cardiac death (first-degree family member died suddenly at age <45 years old in the absence of known heart disease), medical history, physical examination, baseline ECG, results of provocative drug test, EPS when performed and indications for ICD implantation were collected in all patients. Underlying structural cardiac abnormalities were excluded in each patient. Patients presenting with syncope were considered as symptomatic. Syncope was defined as a non-traumatic transient loss of consciousness and spontaneous complete recovery (29). The study was conducted in compliance with the Declaration of Helsinki, approved by the local ethics committee (Comitato Etico per la Sperimentazione Clinica, Azienda Ospedaliera di Padova, Italy) and all patients signed informed consent.

### Electrocardiogram

Baseline 12-lead ECG (speed of 25 mm/s, 1 mV/10 mm gain, and 0.05–150 Hz filter) was recorded in each patient at the first clinical evaluation. The following parameters were recorded in leads II and V6: the RR interval, PQ interval, QRS duration, JT interval, and corrected QT interval. The QTc interval was calculated with the Bazett's formula. In leads V1 to V3, the maximal ST-segment elevation was measured at the J-point (STJ). An electrocardiogram was considered diagnostic of BrS if a coved-type ST-segment elevation of ≥2 mm (Type 1) was documented in ≥1 lead from V1 to V3 positioned in the 2nd, 3rd, or 4th intercostal space, in the presence or absence of a sodium-channel blocker. Conduction disturbances were defined as the presence of at least one of the following conduction abnormalities on basal ECG: first-degree atrio-ventricular (AV) block, prolonged QRS duration, f-QRS, and S-wave in lead I. A QRS interval duration >120 ms was considered prolonged. First-degree AV block was considered in the presence of a PR interval >200 ms. Left bundle branch block, right bundle branch block, left anterior fascicular block, and left posterior fascicular block were defined in accordance with current guidelines (30). Abnormal fragmentation of the QRS complex was defined as the presence of multiple spikes within the QRS complex as described previously (20). The presence of an S-wave ≥ 0.1 mV and/or >40 ms in lead I was examined as described previously (22). Early repolarization pattern was defined as an elevation of the J-point of at least 1 mm above the baseline level, in at least two consecutive inferior (II, III, aVF) or lateral (I, aVL, and V4 to V6) leads either as QRS slurring or notching (2, 23, 24). Two independent experienced electrophysiologists analyzed all the electrocardiograms. In cases of disagreement, a third physician was consulted.

### Electrophysiological study

The EPS included basal measurements of conduction intervals (baseline AH and HV intervals) and programmed ventricular stimulation. The protocol used was at discerption of the center. A maximum of 3 ventricular extrastimuli (with minimum coupling interval of 200 ms) were delivered. A patient was considered inducible if a sustained ventricular arrhythmia, such as VF, VT, or monomorphic VT lasting >30 s or requiring termination because of hemodynamic compromise was induced. Inducibility at EPS was deemed as an indication for ICD implantation (1, 2).

### Follow-up

The primary endpoint of the study was to assess the late arrhythmic outcome defined as a combined endpoint including cardiac arrest/sudden cardiac death (SCD) and appropriate ICD therapy which occurred after GR. Appropriate ICD therapy was defined as an ICD shock delivered in response to VT or VF or anti-tachycardia pacing (ATP) in response to VT and documented by stored intracardiac ECG data during outpatient evaluation or at the remote monitoring. The secondary endpoint was to evaluate a combined endpoint of device-related complications requiring surgical revision and IS after GR. Inappropriate shocks were defined as those delivered in the absence of ventricular arrhythmia.

### Statistical analysis

Categorical variables were described as frequencies (percentages) and differences between groups were evaluated by using the *X*^2^-test or the Fisher exact test as appropriate. Normal distribution of continuous variables was assessed by using the Shapiro-Wilk test. Continuous variables were expressed as mean ± standard deviation (SD) or median (25th−75th percentiles) for normally distributed and skewed variables, respectively, and compared with the Student's *t*-test or the Mann–Whitney *U*-test, as appropriate. The mean event rate per year was evaluated by the number of events occurring during the follow-up divided by the number of patients multiplied by the average duration of follow-up. Survival analysis was performed visually through Kaplan–Meier survival curves, which were later compared using the log-rank test. Patients were censored at the time of the first event or at the time of the last follow-up. Univariate analysis was performed using the Cox proportional hazards model. Variables with a *P* < 0.05 at univariate analysis were entered into the multivariate model. A *p* < 0.05 was considered statistically significant. Statistical analyses were conducted using STATA version 14.1 (STATA Corporation, College Station, TX, USA).

## Results

### Baseline clinical characteristics at enrolment

The study population consisted of 105 patients (79 males; 75%) with a mean age at first ICD implantation of 45 ± 14 years who underwent GR. Ninety-five patients (90.5%) received a TV-ICD and 10 patients (9.5%) an S-ICD. Table 1 shows the clinical characteristics of the study population. At enrollment, no patient had a history of previous cardiac arrest; 61 patients (58%) were symptomatic for syncope, 25 (24%) reported a history of paroxysmal atrial fibrillation (AF) and 44 (42%) were asymptomatic. A spontaneous type 1 ECG was documented in 53 patients (50%). A family history of BrS or SCD was ascertained in 34 (32%) and 48 (46%) patients, respectively. Of 90 patients (86%) undergoing EPS, 52 (58%) were inducible. All patients received a prophylactic ICD because of syncope (*n* = 61), inducibility at EPS (*n* = 52) or both. After the first ICD implantation 30 patients (28.5%) experienced a total of 37 device-related complications including IS (*n* = 6), lead failure (*n* = 9), pocket hematoma (*n* = 8), pocket infection (*n* = 6), pneumothorax (*n* = 3), device dislocation (*n* = 2), systemic infection (*n* = 1), lead dislocation (*n* = 1), cardiac perforation (*n* = 1). The median period from first ICD implantation to last follow-up was 155 (128–181) months and from first ICD implantation to the GR was 84 (61–102) months.

**Table 1:** Clinical and electrocardiographic characteristics of the study population.

**Variables**	**(*****N*** = **105)**
Age at first implantation (years)	45 ± 14
Age at GR (years)	52 ± 14
Male sex, *n* (%)	79 (75)
Family history of BrS, *n* (%)	34 (32)
Family history of SCD, *n* (%)	48 (46)
Syncope, *n* (%)	61 (58)
History of AF, *n* (%)	25 (24)
Positive EPS	52/90 (58)
Basal electrocardiogram	
Spontaneous Brugada type 1, *n* (%)	53 (50)
Early repolarization, *n* (%)	4 (4)
QTc prolongation, *n* (%)	4 (4)
Conduction disturbances, *n* (%)	55 (52)
First degree AV block, *n* (%)	18 (17)
QRS fragmented or prolonged, *n* (%)	13 (12)
S-wave in lead I, *n* (%)	46 (44)

*AF, atrial fibrillation; AV, atrioventricular; BrS, Brugada syndrome; EPS, electrophysiological study; GR, generator replacement; SCD, sudden cardiac death*.

#### Electrocardiographic findings at first clinical evaluation

Table 1 shows the electrocardiographic findings of the study population. Conduction disturbances were found in 55 patients (52%) A first-degree AV block was documented in 18 patients (17%, mean PR duration 221 ± 16 ms). Prolonged QRS duration was observed in 9 subject (8%); 4 (4%) showed a f-QRS in leads V1, V2, V3 while 4 patients (4%) a prolonged QTc interval. Left anterior fascicular block was present in 7 patients (6%). A prominent S-wave in lead I was documented in 46 patients (44%). Early repolarization pattern was present in 4 patients (4%). No patient had a BrS pattern in leads other than V1–V3.

### Generator replacement

No patient experienced major arrhythmic events including appropriate ICD intervention before GR. Reasons for GR were: battery depletion (*n* = 86; 82%), transvenous lead failure (*n* = 9; 8.5%), pocket infection (*n* = 6; 6%), device dislocation/cardiac perforation (*n* = 3, 2.8%) and systemic infection (*n* = 1;1%). Lead failure and infection were indication for transvenous lead extraction. The replacing device was the same type of ICD (TV-ICD or S-ICD) implanted at first in all patients.

### Follow-up after generator replacement

**Figure 1:**
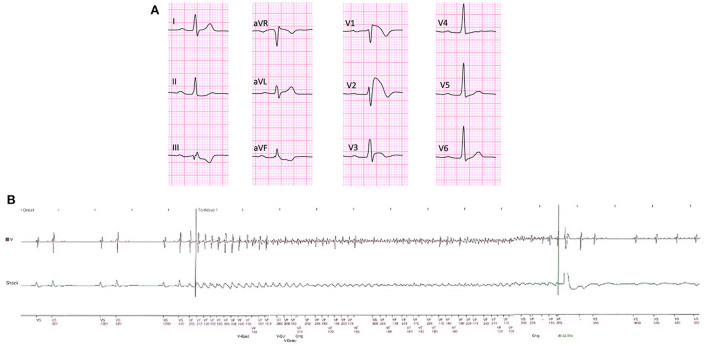
Baseline ECG of an asymptomatic BrS patient who experienced VF 4 years and 6 months after generator replacement. Note the presence of first-degree AV block and S-wave in lead I as well as spontaneous “coved type” ECG pattern in leads V1 and V2 **(A)**. Intracardiac electrocardiogram obtained from the transvenous ICD remote monitoring showing the onset of VF, triggered by a premature ventricular contraction and its offset by the ICD shock **(B)**.

**Figure 2:**
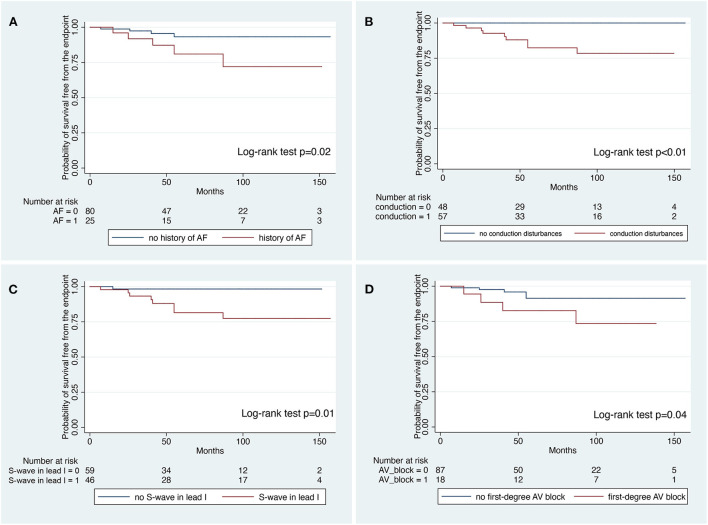
Kaplan–Meier analysis for survival free from the endpoint according to the presence of history of atrial fibrillation **(A)** conduction disturbances, **(B)** S-wave in lead I, **(C)** and first degree atrio-ventricular block **(D)**.

The median follow-up after GR was 57 [38–102] months. Ten patients (9%) received successful >1 appropriate ICD intervention after GR (1.6%/year). The first intervention included shock on VF (*n* = 8 patients), shock on VT (*n* = 1 patient) and ATP on VT (*n* = 1 patient). Appropriate shocks occurred in 6 of the asymptomatic patients (6/44;13.6%; Figure 1). The median time to first appropriate ICD therapy was 108 (101–137) months from the first ICD implantation and 41 [25–55] after GR. Patients who experienced an appropriate ICD intervention had significantly more often a history of paroxysmal AF (*P* = 0.01) compared with patients without appropriate ICD intervention (Table 2). No significant difference between patients who did and did not have arrhythmic events during follow-up with regard to age, gender, family history for BrS or SCD and EPS result was observed. Among the ECG parameters, the presence of conduction abnormalities (*P* = 0.001) and S-wave in lead I (*P* = 0.005) were significantly associated with arrhythmic events (Table 2). At survival analysis, history of AF (log-rank test; *P* = 0.02), conduction disturbances (log-rank test; *P* < 0.01), S wave in lead I (log-rank test; *P* = 0.01) and first-degree AV block (log-rank test; *P* = 0.04) were significantly associated with the occurrence of late appropriate ICD intervention (Figures 2A–D). All patients without conduction disturbances had an uneventful follow-up for appropriate ICD intervention (Figure 2B).

**Table 2:** Baseline clinical and electrocardiographic findings according to late appropriate ICD intervention.

**Variable**	**ICD** ** therapy –** ** (***N* = **95)**	**ICD** **therapy** **+** ** (*****N*** = **10)**	***P*** **-value**
Age at GR (years)	45 ± 14	45 ± 15	0.79
Male sex, *n* (%)	71 (75)	8 (80)	1.00
Family history of BrS, *n* (%)	31 (33)	3 (30)	1.00
Family history of SCD, *n* (%)	43 (45)	5 (50)	1.00
Syncope, *n* (%)	57 (60)	4 (40)	0.31
History of AF, *n* (%)	19 (20)	6 (60)	0.01
Positive EPS	46 (55)	6 (86)	0.53
Basal electrocardiogram			
Spontaneous Brugada type 1, *n* (%)	48 (51)	5 (50)	1.00
Early repolarization, *n* (%)	3 (3)	1 (10)	0.33
QTc, *n* (%)	3 (3)	1 (10)	0.33
Conduction disturbances, *n* (%)	45 (47)	10 (100)	0.001
First degree AV block, *n* (%)	14 (15)	4 (40)	0.06
QRS fragmented or prolonged, *n* (%)	10 (10)	3 (30)	0.10
S-wave in lead I, *n* (%)	37 (39)	9 (90)	0.005

*AF, atrial fibrillation; AV, atrioventricular; BrS, Brugada syndrome; EPS, electrophysiological study; GR, generator replacement; SCD, sudden cardiac death*.

After GR replacement 7 patients (6.6%) experienced a total of 12 device-related complications (1.7%/year) including IS (*n* = 3), lead failure (*n* = 4), pocket hematoma (*n* = 1), pocket infection (*n* = 1), and device dislocation (*n* = 3). Among patients with an S-ICD, 2 (20%) experienced IS. Univariate predictors of late arrhythmic events included a history of AF (HR: 4.11; 95% CI: 1.15–14.78; *P* = 0.03) and S-wave in lead I (HR: 10.12; 95% CI: 1.28–79.97; *P* = 0.02) (Table 3). In the multivariate model, only S-wave in lead I remained a significant independent predictor of late arrhythmic outcome (HR: 9.17; 95% CI: 1.15–73.07; *P* = 0.03) (Table 3).

**Table 3:** Univariate and multivariate cox regression analysis for predictors of late appropriate ICD intervention.

**Variable**	**Univariate analysis**		**Multivariate analysis**	
	**HR (95% CI)**	***P*** **-value**	**HR (95% CI)**	***P*** **-value**
Age <50 years-old at GR	1.21 (0.32–4.51)	0.78		
Male sex	1.38 (0.28–6.68)	0.69		
Family history of BrS	0.93 (0.23–3.75)	0.93		
Family history of SD	1.16 (0.34–4.02)	0.81		
Syncope	0.52 (0.15–1.86)	0.32		
History of AF	4.11 (1.15–14.78)	0.03	3.68 (0.98–13.63)	0.06
Positive EPS	1.12 (0.31–4.20)	0.86		
Spontaneous Brugada type 1	0.93 (0.27–3.24)	0.92		
QTc prolongation	1.15 (0.11–12.34)	0.91		
Early repolarization	3.54 (0.43–28.82)	0.24		
Conduction disturbances^*^	3.33 (0.89–12.45)	0.07		
First-degree AV block				
QRS fragmentated or prolonged	6.54 (0.79–53.93)	0.08		
S-wave in lead I	10.12 (1.28–79.97)	0.02	9.17 (1.15–73.07)	0.03

AF, atrial fibrillation; AV, atrioventricular; BrS, Brugada syndrome; EPS, electrophysiological study; GR, generator replacement; SCD, sudden cardiac death.

* Cox regression could not be performed because no primary endpoint events occurred in patients without conduction disturbances.

## Discussion

The aim of the present long-term multicenter study was to assess the incidence and clinical-electrocardiographic predictors of late life-threatening arrhythmias in BrS patients treated with prophylactic ICD and undergoing GR. The main findings are the following: (1) over a long-term follow-up, the risk of late appropriate ICD therapy after GR remains clinically relevant in up to 9% of patients; (2) late arrhythmic events during follow-up were significantly associated with a history of AF and conduction abnormalities detected at baseline clinical evaluation (first-degree AV block and S-wave in lead I); (3) at multivariate analysis the presence of S-wave in lead I remained the only independent predictor of life-threatening arrhythmias. These findings support the need for GR at the end of service regardless of previous appropriate intervention, mostly in BrS patients with conduction abnormalities.

Risk stratification in BrS remains a clinical challenge. According to previous studies, history of cardiac arrest or syncope are the strongest predictors of SCD (4–10). The prognostic value of a history of familial SCD (1, 2), positive genetic testing for a SCN5A-gene mutation (1, 2) and history of AF (31) is less well-established. It is known that SCN5A-gene mutation is a major contributor of BrS. However, only 20–30% of BrS patients carry mutations of SCN5A (1). Recently published data reported that other mutations, including SCN1B, SCN10A, and SNTB2 are also associated with BrS. Specific genetic mutations have been also related to specific genotype–phenotype association, including cardiac conduction dysfunction, AF, ventricular arrhythmias and susceptibility to sodium channel blockers (32–35). These findings may provide new opportunities to further elucidate the cellular disease mechanism of BrS, improve screening, and risk stratification.

ECG abnormalities both depolarization and repolarization, such as ER, increased QRS duration (19), f-QRS (20, 21), first degree AV block (18), and S-wave in lead I (22, 36) have been associated with a worse outcome. The role of EPS remains controversial (1, 2, 7–11). Our study reported that S-wave in lead I is an independent predictor of late arrhythmic events. Accordingly, the presence of this ECG abnormality should be added to the list of the ECG variables predicting a worse outcome in BrS who received a prophylactic ICD implantation.

### ICD therapy in Brugada syndrome

To date, ICD remains the only therapy with proven efficacy in preventing SCD in BrS patients (1–3, 25–27). However, ICD implantation is not risk-free being associated with high rates of IS and device-related complications (25–27). Increase in diagnosis of patients with BrS has led to an increase of ICD implantations (1, 2). The decision to implant an ICD is not without risks given that up to 24% of BrS patients experience IS (25). Moreover, multiple GR procedures may be needed, with a potential increased rate of device-related complications, ranging from 15.9 to 36% (25–27). It is controversial whether device replacement is needed in patients who never experienced appropriate ICD therapy until the time of GR. Considering the ICD complications and cost, ICD replacement in patients without previous appropriate therapy should be evaluated carefully. Thus, the balance between the potential life-saving and the risk of complications after ICD replacement in asymptomatic BrS patients and without appropriate ICD intervention before GR remains to be established. Of note, in our study, appropriate shocks occurred in 13% of asymptomatic patients which is in line with previous long-term studies (25, 26). In our study, after ICD replacement ≥1 device-related complications requiring surgical revision occurred in 6.6% of patients.

A prior study by Kim et al. (37) reported the potential benefit of ICD therapy after GR in a small cohort of patients with BrS treated for either primary or secondary prevention. Our multicenter study confirmed and extended these previous observations by demonstrating in a large BrS population who received prophylactic ICD and did not experience appropriate ICD therapy, that the risk of late appropriate ICD therapy after GR remains significant in a clinically relevant proportion of cases, mostly in the presence of conduction abnormalities such as S-wave in lead I on basal ECG. According to the results of the present study, S-wave in lead I on basal ECG, may contribute to the accurate analysis of risk-benefit ratio when considering GR in BrS who received a prophylactic ICD implantation.

Subcutaneous implantable cardioverter-defibrillator is an alternative option to TV-ICD therapy to reduce lead-related complications (38). However, S-ICD in patients with BrS is associated with relatively high risk rate of IS (39), if one considers that in our study 2 patients of 10 patients (20%) with an S-ICD experienced IS due to signal oversensing. Most important, a sizeable proportion of patients with BrS are not eligible to S-ICD because they fail the pre-implantation screening (40).

### Conduction abnormalities in Brugada syndrome

Although the potential prognostic role of the presence of different types of conduction abnormalities in BrS has been reported (18–22, 36), none of ECG conduction abnormalities are currently used for risk stratification. Recently, our study group, for the first time, demonstrated that first-AV block was an independent predictor of malignant arrhythmic events (18). A more recent meta-analysis by Pranata et al. confirms that first-degree AV block is associated with more frequent major arrhythmic events in BrS patients (41). In the present study, we found that among ECG abnormalities, patients who experienced malignant arrhythmic events after GR had more often conduction abnormalities including first-degree AV block at baseline (*P* = 0.04). At variance with our results, fQRS has been linked to poor prognosis in previous studies (16, 17). In a prospective study on 347 consecutive patients with BrS with spontaneous type 1 ECG pattern and no history of cardiac arrest, Calò et al. (22) found that the presence of an S-wave in lead I was a predictor of life-threatening ventricular arrhythmias. The potential prognostic value of this ECG conduction parameter was strengthened in a more recent study by Giustetto et al. (36) extending it to patients with drug-induced Type 1 ECG and patients with previous cardiac arrest. According to our results the presence of S-wave in lead I on basal ECG is an independent predictor of ventricular arrhythmias. S-wave in lead I may be due to prolongation of the QRS complex, expression of a conduction delay localized in the right ventricular outflow tract (RVOT) (22). To this regard, several studies investigated the arrhythmogenic substrate in BrS patients and found that conduction abnormalities in the RVOT may represent a possible underlying arrhythmogenic substrate (17).

The results of the present and some previous studies confirmed that patients with BrS may exhibit variable degree of conduction abnormalities. Recently Migliore et al. found coexistence of Brugada repolarization abnormalities and conduction disturbances in a 35-year-old man who died suddenly. The histological examination demonstrated severe disruption by fibrous tissue of the proximal tract of both right and left bundle branches (18) suggesting the presence of underlying structural heart abnormalities in BrS. We can speculate, that the presence of underlying conduction disturbances associated with aging of the conduction system and drug interference on the conduction system itself could increase the arrhythmic risk in patients with BrS.

### Study limitations

Limitations are present in our study. This is a retrospective multicenter study. Even with this very long follow-up there was a relatively small number of malignant arrhythmic events, and this might have affected the identification of unique predictors on multivariate analysis. Most of the ECG parameters analyzed in the study are dynamic, and the real prevalence of these parameters is difficult to evaluate. Moreover, EPS protocol and ICD implantation procedures were performed at discretion of the center protocol and physician. Finally, only few patients underwent genetic testing and no analysis combining genotype and arrhythmic risk was performed because, by study design, it was not a genotype–phenotype correlation study.

## Conclusions

The present study indicates that BrS patients receiving a prophylactic ICD may experience late appropriate intervention after GR in a clinically relevant proportion of cases. S-wave in lead I on basal ECG at the time of first clinical evaluation was the only independent predictor of persistent risk of life-threatening arrhythmic events. These findings support the need for GR at the end of service regardless of previous appropriate intervention, mostly in BrS patients with conduction abnormalities.

## Data availability statement

The raw data supporting the conclusions of this article will be made available by the authors, without undue reservation.

## Ethics statement

The studies involving human participants were reviewed and approved by Local Ethics Committee: Comitato Etico per la Sperimentazione Clinica, Azienda Ospedaliera di Padova, Italy. The patients provided their written informed consent to participate in this study.

## Author contributions

FM, NM, LC, and DC contributed to conception and design of the study. FM, NM, AM, GW, RV, CC, AR, LP, VM, JS, G-BC, ACurc, GA, RM, FS, ACurn, EB, and MD organized the database. RV and AZ performed the statistical analysis. FM, CA, LP, and DC wrote the first draft of the manuscript. FM, NM, LP, CA, and DC wrote the sections of the manuscript. All authors contributed to manuscript revision, read, and approved the submitted version.

## Conflict of interest

VM received an educational grant from the Enrico and Enrica Sovena Fundation, Italy. G-BC received compensation for teaching purposes and proctoring from Medtronic, Abbott, Biotronik, Boston Scientific, Acutus Medical. CA receives research grants on behalf of the center from Biotronik, Medtronic, Abbott, LivaNova, Boston Scientific, AtriCure, Philips, Acutus, and received compensation for teaching purposes and proctoring from Medtronic, Abbott, Biotronik, Livanova, Boston Scientific, Atricure, Acutus Medical, and Daiichi Sankyo. The remaining authors declare that the research was conducted in the absence of any commercial or financial relationships that could be construed as a potential conflict of interest.

## Publisher's note

All claims expressed in this article are solely those of the authors and do not necessarily represent those of their affiliated organizations, or those of the publisher, the editors and the reviewers. Any product that may be evaluated in this article, or claim that may be made by its manufacturer, is not guaranteed or endorsed by the publisher.
