# Corrigendum: Predictors of late arrhythmic events after generator replacement in Brugada syndrome treated with prophylactic ICD

**DOI:** 10.3389/fcvm.2022.1076294

**Published:** 2022-11-15

**Authors:** Federico Migliore, Nicolò Martini, Leonardo Calo', Annamaria Martino, Giulia Winnicki, Riccardo Vio, Chiara Condello, Alessandro Rizzo, Alessandro Zorzi, Luigi Pannone, Vincenzo Miraglia, Juan Sieira, Gian-Battista Chierchia, Antonio Curcio, Giuseppe Allocca, Roberto Mantovan, Francesca Salghetti, Antonio Curnis, Emanuele Bertaglia, Manuel De Lazzari, Carlo de Asmundis, Domenico Corrado

**Affiliations:** ^1^Department of Cardiac, Thoracic, Vascular Sciences and Public Health, University of Padova, Padova, Italy; ^2^Department of Cardiology, Policlinico Casilino, Rome, Italy; ^3^Heart Rhythm Management Centre, Postgraduate Program in Cardiac Electrophysiology and Pacing, Universitair Ziekenhuis Brussel-Vrije Universiteit Brussel, European Reference Networks Guard-Heart, Brussels, Belgium; ^4^Division of Cardiology, Department of Medical and Surgical Sciences, Magna Graecia University, Catanzaro, Italy; ^5^Department of Cardiology, S.Maria dei Battuti Hospital, Conegliano, Italy; ^6^Spedali Civili Hospital, University of Brescia, Brescia, Italy

**Keywords:** Brugada syndrome, implantable cardioverter-defibrillator, risk stratification, sudden cardiac death, complications

In the published article, there was an error in Table 3 (Univariate and multivariate cox regression analysis for predictors of late appropriate ICD intervention). The values “9.17 (1.15–73.07) 0.03” of the multivariate analysis were incorrectly reported in the line of the variable “First-degree AV block.” The correct alignment is at the level of the variable “S-wave in lead I.” The corrected Table 3 and its caption appear below.

**Table 3:** Univariate and multivariate cox regression analysis for predictors of late appropriate ICD intervention.

**Variable**	**Univariate analysis**		**Multivariate analysis**	
	**HR (95% CI)**	***P*** **-value**	**HR (95% CI)**	***P*** **-value**
Age <50 years-old at GR	1.21 (0.32–4.51)	0.78		
Male sex	1.38 (0.28–6.68)	0.69		
Family history of BrS	0.93 (0.23–3.75)	0.93		
Family history of SD	1.16 (0.34–4.02)	0.81		
Syncope	0.52 (0.15–1.86)	0.32		
History of AF	4.11 (1.15–14.78)	0.03	3.68 (0.98–13.63)	0.06
Positive EPS	1.12 (0.31–4.20)	0.86		
Spontaneous Brugada type 1	0.93 (0.27–3.24)	0.92		
QTc prolongation	1.15 (0.11–12.34)	0.91		
Early repolarization	3.54 (0.43–28.82)	0.24		
Conduction disturbances^*^				
First-degree AV block	3.33 (0.89–12.45)	0.07		
QRS fragmentated or prolonged	6.54 (0.79–53.93)	0.08		
S-wave in lead I	10.12 (1.28–79.97)	0.02	9.17 (1.15–73.07)	0.03

AF, atrial fibrillation; AV, atrioventricular; BrS, Brugada syndrome; EPS, electrophysiological study; GR, generator replacement; SCD, sudden cardiac death.

* Cox regression could not be performed because no primary endpoint events occurred in patients without conduction disturbances.

The authors apologize for this error and state that this does not change the scientific conclusions of the article in any way. The original article has been updated.

## Publisher's note

All claims expressed in this article are solely those of the authors and do not necessarily represent those of their affiliated organizations, or those of the publisher, the editors and the reviewers. Any product that may be evaluated in this article, or claim that may be made by its manufacturer, is not guaranteed or endorsed by the publisher.

